# Editorial: Molecular Biomarkers for Gastric Cancer

**DOI:** 10.3389/fonc.2022.850373

**Published:** 2022-02-03

**Authors:** Xinqiang Hong, Fenglin Liu

**Affiliations:** Department of General Surgery, Zhongshan Hospital, Fudan University, Shanghai, China

**Keywords:** molecular biomarkers, gastric cancer, bioinformatic technology, prognostic biomarker, therapeutic target

Gastric cancer (GC) is one of the leading causes of malignancy and tumor-related death worldwide, especially in Eastern countries (1). Since gastric cancer is often asymptomatic in its early stages, non-invasive methods for early detection are urgently required. The ideal biomarkers should facilitate early diagnosis, accurate prognosis, timely detection of recurrence or metastasis during postoperative surveillance and guidance for individualized treatment. Classical tumor biomarkers such as carcinoembryonic antigen (CEA) and cancer antigen 19-9 (CA19-9), which are commonly used in clinical practice, have limited specificity and sensitivity (2). Moreover, gastric cancer is a highly heterogeneous disease, but there are still many shortcomings in its molecular typing and individualized treatment. HER2 was the first molecular biomarker to be put into clinical use, accompanied by the first molecular targeted drug being included in gastric cancer treatment guidelines, trastuzumab (3). However, the main problems facing trastuzumab are the low proportion of gastric cancer patients overexpressing HER2 and drug resistance. Disappointingly, several subsequent clinical studies of other drugs targeting HER2 have failed to achieve satisfactory results (4, 5).

With the rapid development of bioinformatic technology, we can obtain information on gene expression and gene mutations in gastric cancer from large databases such as The Cancer Genome Atlas (TCGA) and Gene Expression Omnibus (GEO), and screen out differentially expressed genes that are relevant to diagnosis or prognosis for further analysis. Zhang et al. extracted somatic mutation data from the TCGA Stomach Adenocarcinoma (STAD) cohort and developed a four-gene-based risk score significantly associated with overall survival prognosis, and validated it in the Tianjin cohort. Based on the analysis of TCGA database, Li et al. indicated several possible key genes related to gastric cancer progression and prognosis, including TP53, HRAS, BRCA1, PIK3CA, AKT1, and SMARCA4. Chen et al. constructed and validated a prognostic nomogram model based on 5 DNA methylation-driven genes (CXCL3, F5, GNAI1, GAMT and GHR) for gastric cancer, promising as a valid prognostic assessment tool. Shan et al. detected differentially expressed genes between gastric cancer and adjacent normal tissues using gene expression profiling datasets from the Gene Expression Omnibus (GEO) database, and three genes (CDH3, LEF1 and MMP7) were then screened as possible predictors of gastric cancer prognosis by bioinformatics methods. Based on information from the TCGA database, Xie et al. showed that high PAFAH1B3 (Platelet activating factor acetylhydrolase 1b catalytic subunit 3) expression in gastric cancer was significantly associated with proliferation-related gene sets and with immune cell infiltration, and *in vitro* experiments verified that its function involves cell proliferation, migration and the activation of oncogenic signaling. Zhang et al. combined data from 5 pairs of gastric cancer samples and TCGA, and found that DDX18 expression correlated with tumor volume, Borrmann classification, degree of tumor differentiation, cancer embolus, lymph node metastasis and TNM stage, suggesting it as a prognostic biomarker. The authors also explored the mechanism of DDX18 *in vitro* and *in vivo*, suggesting that it could be a potential therapeutic target as well. Taking advantage of TCGA and GEO databases, Ye et al. constructed A 13-gene metabolic signature (GSTA2, POLD3, GLA, GGT5, DCK, CKMT2, ASAH1, OPLAH, ME1, ACYP1, NNMT, POLR1A and RDH12) that can be used for the prognosis of gastric adenocarcinoma and suggests its relationship with the immune microenvironment. Huo et al. obtained data from the TCGA database, identified seven differentially expressed immune-related genes based on tumor-associated macrophage infiltration, constructed a risk scoring system that predicted gastric cancer prognosis, and validated it in four cohorts of the GEO database. Tan et al. identified five differentially expressed genes (MS4A1, THBS2, VCAN, PDGFRB, and KCNA3), among which VCAN and PDGFRB were associated with poor prognosis and their functions may involve extracellular matrix and receptor ligand activity, based on data of early-stage stomach adenocarcinoma from TCGA. Based on the information obtained from the TCGA database, Li et al. discovered that the GUCY1A2 gene (encoding the soluble guanylyl cyclase α2 subunit) was highly expressed in gastric cancer tissues and was associated with histological grade and T stage, and its high expression predicted a poor prognosis. Ma et al. utilized TCGA and Immport database to derive an immune-related lncRNA signature and validated it in 75 gastric cancer samples, demonstrating its capacity to predict overall survival and immune status of the tumor microenvironment in gastric cancer.

Additionally, some authors have explored and studied biomarkers associated with the diagnosis, treatment efficiency and prognosis of gastric cancer using data of gastric cancer patients from their own institutions. Guo et al. found that combined analysis of large tumor suppressor kinases 1/2 (LATS1/2), CD8, and FOXP3 has a prognostic value for gastric cancer. The effect of LATS1/2 on CD8^+^ T cells and POXP3^+^ Treg deserves further investigation and is expected to provide potential targets for immunotherapy. Kim et al. identified a GC-specific radiosensitivity gene signature by analyzing 12 gastric cancer cell lines and screened that the Akt pathway was most associated, suggesting that it could be used as a target for radiotherapy sensitization. Song et al. demonstrated that human hedgehog-interacting protein (HHIP) regulates gastric cancer progression and metastasis by regulating promoter methylation levels, and its lower level is positively correlated with gastric cancer metastasis, which is expected to be a prognostic marker and therapeutic target. Wang et al. analyzed 322 surgical specimens of early gastric cancer that had undergone radical gastrectomy and found that tumor-associated neutrophils (TANs) were associated with tumor volume, depth of invasion, Lauren classification, histological classification, lymphovascular invasion and perineural invasion, and were predictive of lymph node metastasis in early gastric cancer. Tong et al. revealed that high expression of tRF-3017A (derived from tRNA-Val-TAC) in gastric cancer tissues was associated with lymph node metastasis, and mechanistic studies suggested that it could promote migration and invasion of gastric cancer cells by silencing the cancer suppressor genes NELL2. Xu et al. examined 155 gastric cancer specimens and found that high PCSK9 (proprotein convertase subtilisin/kexin type 9) expression was associated with GC progression and poor prognosis. *In vitro* and *in vivo* assays validated that PCSK9 could promote GC metastasis and suppress apoptosis by facilitating MAPK signaling pathway through HSP70 up-regulation. Cai et al. analyzed a cohort of patients with stage III gastric cancer and concluded that microsatellite instability-high (MSI-H) had a poorer response to neoadjuvant chemotherapy but better survival compared to microsatellite stability or microsatellite instability-low (MSS/MSI-L), requiring further investigation about the value of neoadjuvant chemotherapy for Stage III GC patients with MSI-H. Ye et al. demonstrated that high US31 expression in gastric cancer patients predicted better overall survival. US31 overexpression inhibited gastric cancer cell proliferation, migration and invasion *in vitro*, and it was also involved in regulating the tumor immune microenvironment, may playing the dual role as prognostic biomarker and therapeutic target. Zhang et al. showed that lncRNA SNHG12 is highly expressed in gastric cancer cells and tissues, predicting a poor prognosis. The authors also performed *in vitro* and *in vivo* experiments to confirm its association with cell proliferation by regulating the YWHAZ/AKT/GSK-3β axis. Wang et al. constructed a novel scoring system called the inflammatory-nutritional prognostic score (INPS) based on data from 513 patients with stage III gastric cancer, which was a valid independent prognostic factor for patients receiving radical gastrectomy followed by adjuvant chemotherapy. Zhang et al. analyzed data of 166 gastric cancer patients and noted that high expression of leukocyte immunoglobulin like receptor subfamily B1 (LILRB1), thought to be an immunosuppressive molecule, was associated with more advanced tumor stage, higher risk of recurrence and poor survival, and that it was positively correlated with M2 tumor-associated macrophages (TAMs) infiltration, which could produce an immunosuppressive microenvironment. Finally, there is a meta-analysis conducted by Wei et al. that included 12 studies with 8,305 patients and showed that a low albumin-to-globulin ratio (AGR) prior to treatment for gastric cancer predicted worse overall survival (OS) and disease-free survival/progression-free survival (DFS/PFS).

In summary, a lot of studies are trying to find effective biomarkers or scoring systems to help early diagnosis, efficacy prediction and evaluation, monitoring of recurrence and metastasis, and individualized treatment for gastric cancer, as well as to deepen our understanding of the pathogenesis of gastric cancer. However, there are several challenges in translating these findings to clinical applications. First, biomarkers for early diagnosis need to be tested in a large population including healthy individuals to clarify sensitivity and specificity. Secondly, most biomarkers need to be tested on tumor specimens, which are somewhat invasive and sometimes inaccessible. Moreover, the specimens obtained by endoscopic biopsy may not be an accurate representation of the overall disease due to the heterogeneity of the tumor. In addition, for biomarkers involved in prognosis and therapeutic efficacy prediction, it would be more convincing if investigators employed prospective studies for validation. Finally, we hope that we can improve the journey of gastric cancer biomarkers from discovery, validation to successful clinical translation based on the close cooperation of multidisciplinary teams, so as to achieve earlier detection and more appropriate individualized treatment (6), thus improving the prognosis of gastric cancer.

## Author Contributions

All authors listed have made a substantial, direct, and intellectual contribution to the work and approved it for publication.

## Conflict of Interest

The authors declare that the research was conducted in the absence of any commercial or financial relationships that could be construed as a potential conflict of interest.

## Publisher’s Note

All claims expressed in this article are solely those of the authors and do not necessarily represent those of their affiliated organizations, or those of the publisher, the editors and the reviewers. Any product that may be evaluated in this article, or claim that may be made by its manufacturer, is not guaranteed or endorsed by the publisher.
